# Paget's Presentation of High-Grade Ductal Carcinoma In Situ (DCIS) in a Very Young Female With Breast Cancer 2 (BRCA2) Mutation

**DOI:** 10.7759/cureus.54678

**Published:** 2024-02-22

**Authors:** Sneha Butala, Chancée Forestier, Sugeetha Nithiananthan, Asilis J Defran, Nafisa K Kuwajerwala

**Affiliations:** 1 Pediatrics, Rainbow Babies & Children's Hospital, Cleveland, USA; 2 Medicine, American University of the Caribbean School of Medicine, Cupecoy, SXM; 3 Obstetrics and Gynecology, Ascension Providence Hospital, Southfield, USA; 4 Surgery, Ascension Providence Hospital, Southfield, USA; 5 General Surgery, Ascension Providence Hospital, Southfield, USA; 6 Breast Surgery, Ascension Providence Hospital, Southfield, USA; 7 Breast Surgery, Ascension Rochester Hills, Rochester Hills, USA; 8 Breast Surgery, Corewell Health, Troy, USA

**Keywords:** paget's disease, ductal carcinoma in situ, breast cancer surgery, breast surgery, oncological surgery, brca gene mutation, breast cancer

## Abstract

This is a case of a previously healthy 29-year-old female with erythema and skin excoriations of the left breast nipple-areolar complex (NAC). After a repeat trial and failure of topical hydrocortisone, a diagnostic mammogram and nipple biopsy revealed Paget's disease (PD) of the nipple with ductal carcinoma in situ (DCIS). A subsequent genetic analysis found a breast cancer 2 (*BRCA2*) gene mutation. Treatment consisted of a left breast skin-sparing simple mastectomy with sentinel lymph node (SLN) biopsy and immediate tissue expander placement for implant reconstruction. Further management involved right breast short-interval surveillance with annual mammography and magnetic resonance imaging (MRI) with the possibility of prophylactic surgery along with oophorectomy after childbearing.

## Introduction

Globally, breast cancer remains the most commonly diagnosed cancer in females and the second most common cause of death in females [1]. Specifically, Paget's disease (PD) of the breast is a rare disease cited as having an incidence of 1%-3% of all primary breast cancers and is almost always associated with underlying carcinoma [1,2]. Paget's disease (PD) of the breast can be subdivided into three subclasses: PD without concurrent breast cancer, PD with ductal carcinoma in situ (PD-DCIS), and PD with invasive ductal carcinoma (PD-IDC). When comparing DCIS to PD-DCIS and IDC to PD-IDC, the breast cancers with associated PD had a more aggressive clinical course, as well as a poorer prognosis compared to DCIS alone or IDC alone [3]. Though Paget's disease of the breast is rare among breast cancers, its importance lies in its less favorable outcomes.

Sir James Paget first described the disease in a report in 1874, identifying 15 patients with nipple-areolar complex (NAC) changes followed by an underlying malignancy in the mammary tissue. These changes were noted as "intensely red, raw surface(s), very finely granular, as if nearly the whole epidermis was removed (on nipple-areolar complex)" [4]. As such, Paget's disease typically presents as a rash or an excoriated lesion of the NAC. However, it may also manifest solely as a pruritic, normal-appearing nipple, making the diagnosis difficult or overlooked [4]. Paresthesias, such as burning and pruritus, may present before any visual signs develop. Therefore, Paget's disease should be considered as a differential diagnosis for patients with these complaints [5]. Due to its presentation similarities to other diseases, Paget's disease can be mistaken for eczema, psoriasis, or dermatitis, leading to the late diagnosis and treatment of the cancer [2]. Therefore, to avoid misdiagnosis and delayed treatment, patients with these suspicious signs should undergo a biopsy in order to rule out Paget's disease [2].

Although Paget's disease is known to mainly present in the sixth decade of life, very few reports have been presented in the literature as occurring in females as young as 24 years of age [4]. Often, when a female presents with complaints of NAC pruritus or eczematous changes, she is initially prescribed topical treatments. Both symptoms are typically seen in younger patients who have no family history of breast malignancy. Breast cancer (*BRCA*) mutation is suspected in very young patients with breast cancer (age < 45) or patients with a significant family history of breast, ovarian, pancreatic, and prostate cancers or melanoma or Hodgkin's lymphoma. As such is the case we present of a 29-year-old female who was referred to our breast cancer center due to NAC erythema and changes in a failed topical treatment regimen and has no family history of cancers in two generations.

## Case presentation

A healthy 29-year-old female began to notice erythema and skin excoriations of the left breast NAC approximately two months prior to being seen by a breast surgeon at our clinic. The patient described the skin changes as cyclical and associated with mastalgia, without any breast lumps.

**Figure 1:**
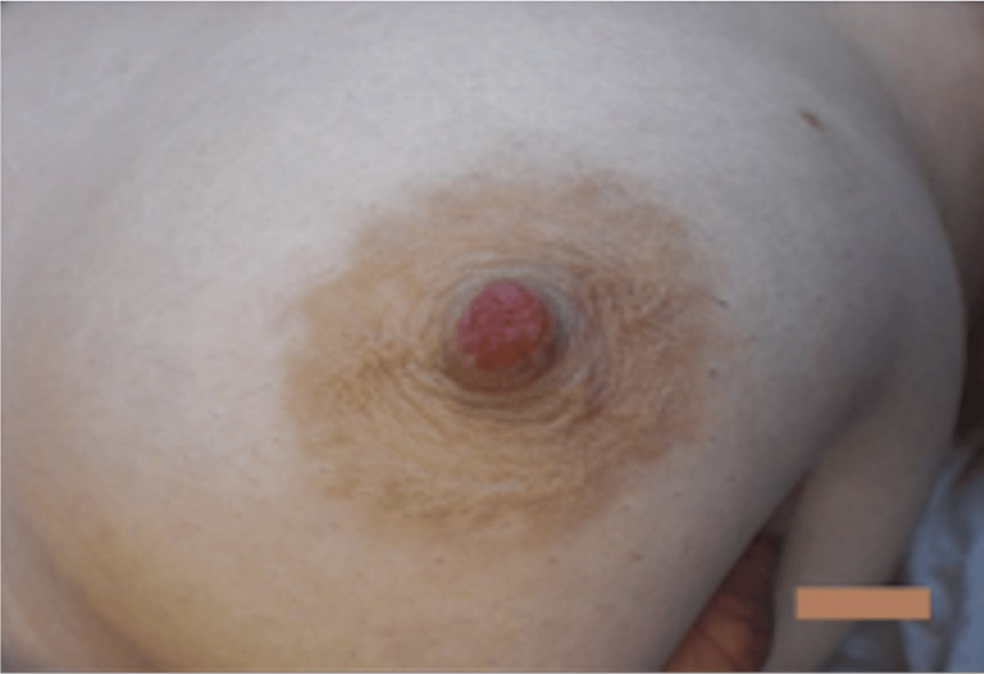
Erythematous and blistered appearance of the left NAC. Image taken during initial presentation at our clinic. NAC: nipple-areolar complex

Clinical examination at our breast care center revealed a healthy, comfortable female with no family history of breast cancer or high-risk factors. Bilateral breast examination failed to reveal axillary, supraclavicular, or cervical lymphadenopathy. No dominant masses or retractions were noted in both supine and upright positions. Bilateral NAC displayed signs of dryness. In addition, the left nipple was erythematous in appearance and best described as blistered (Figure 1).

**Figure 2:**
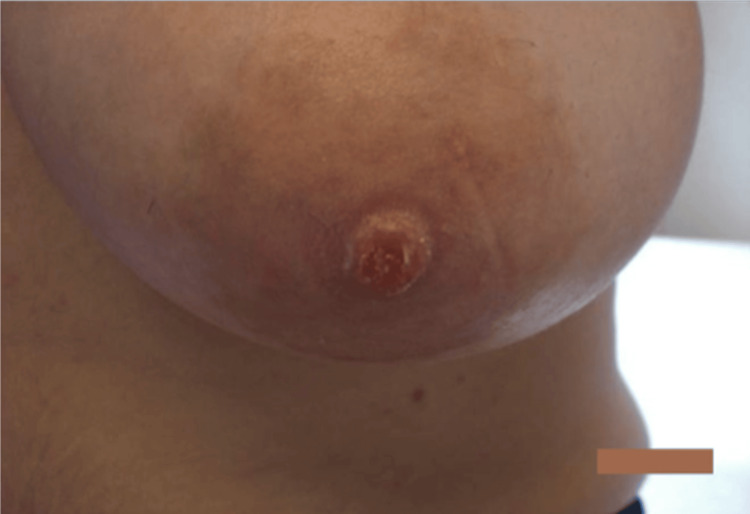
Mild eczematous changes on the left nipple after topical treatment regimen.

In conjunction with a dermatologist, our breast surgeon prescribed daily application of topical hydrocortisone and mupirocin ointment for two weeks. These are often prescribed when a bacterial infection is on the differential. When the patient returned to the clinic one month later, the rash resolved on her areola but persisted on her nipple, as can be seen in Figure 2. Due to the discernable improvement, she was continued on the topical treatments with close surveillance for an additional two months. Throughout the treatment, our patient denied any history of bloody nipple discharge. Her only complaints were cyclical breast pain and the abovementioned NAC changes.

After two months of surveillance, the topical hydrocortisone failed in treating the changes of her NAC, and we encouraged the patient to proceed with breast imaging along with a nipple biopsy as our next diagnostic steps. When considering imaging, ultrasound and mammography are common diagnostic tools used with breast abnormalities. Since this patient's only signs and symptoms were nipple lesion with ulceration and no masses were palpable, a mammogram was recommended over an ultrasound. The patient agreed to undergo bilateral mammography. However, the patient initially felt hesitant to undergo biopsy because of the patient's young age, lack of family history of breast cancer diagnosis, and benign initial consultation. The patient perceived a nipple biopsy as a very invasive diagnostic procedure. Even though she was reluctant, she agreed to a formal biopsy as well.

**Figure 3:**
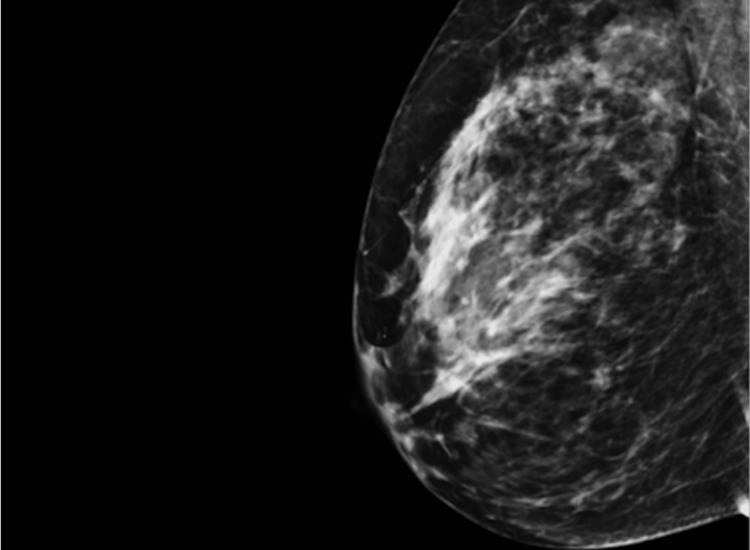
Left breast mammogram MLO view showing dense breast and diffuse calcifications. MLO: mediolateral oblique

**Figure 4:**
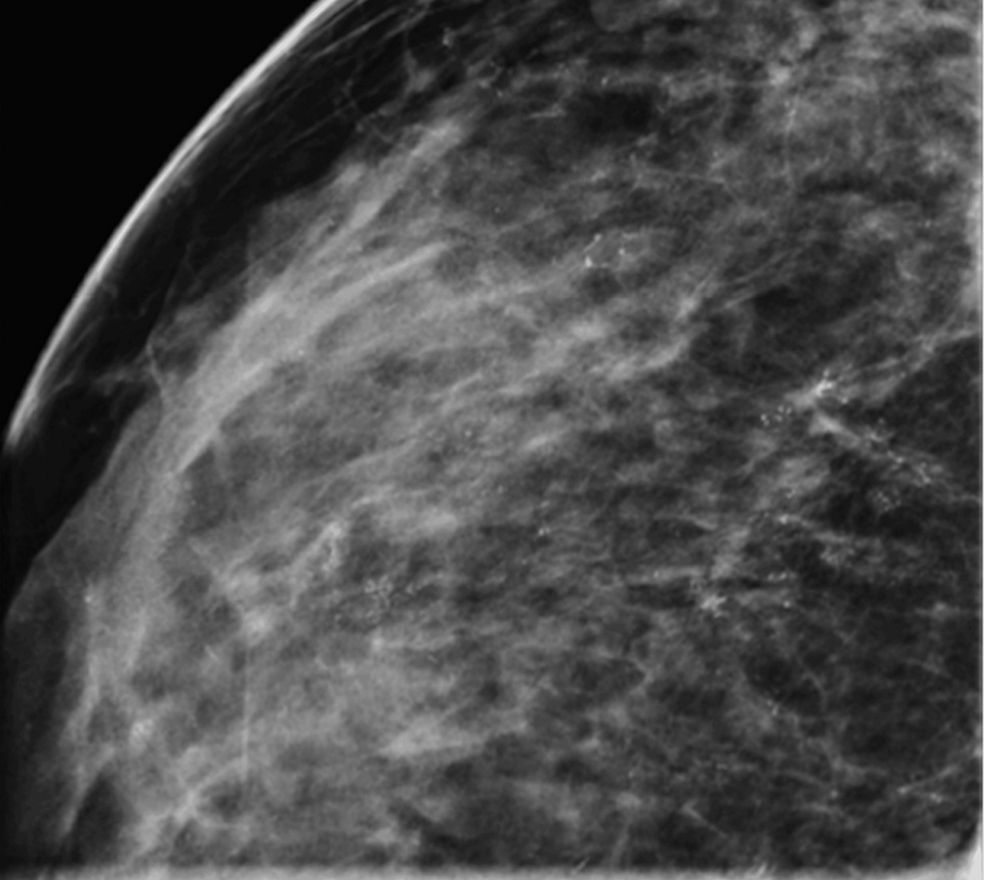
Left breast mammogram magnification of CC lateral view showing dense breast and diffuse calcifications. CC: craniocaudal

The mammography revealed multiple groups of indeterminate calcifications scattered throughout her left breast (Figures 3, 4) without an evidence of an abnormality of the right breast. The findings of the left breast were felt to be highly suspicious, and our consulting radiologist recommended proceeding with a two-site stereotactic core biopsy. Pathology diagnosed high-grade ductal carcinoma in situ (DCIS) of both sites more than 10 cm apart with intervening suspicious calcifications and one specimen also containing a small focal suspicion of microinvasion.

**Figure 5:**
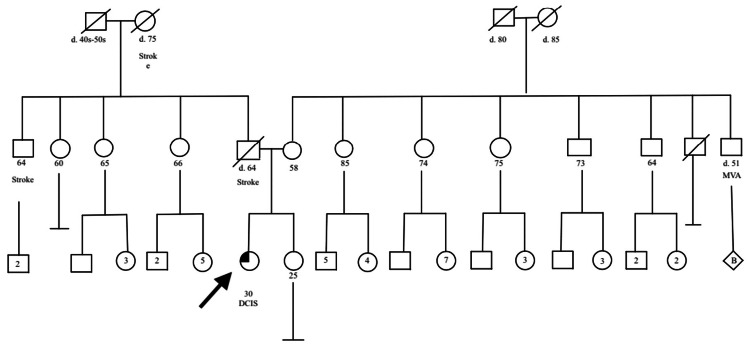
Pedigree of the patient's family. DCIS, ductal carcinoma in situ; MVA, motor vehicle accident

**Figure 6:**
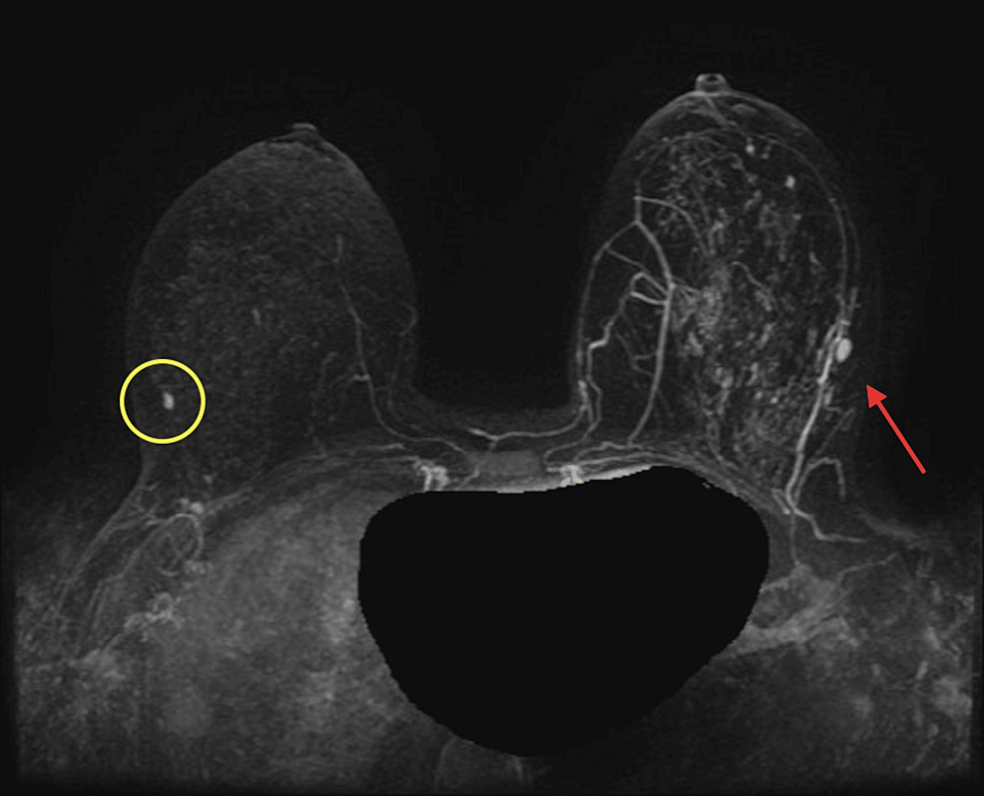
MRI of bilateral breasts showing clumped nodular enhancement in the left breast (arrow), with a probable lymph node in the right breast (circle) MRI: magnetic resonance imaging

Given our patient's young age of 29 years with a diagnosis of high-grade DCIS, estrogen-receptor (ER) and progesterone receptor (PR) negative, clinical stage 0, and noninvasive (Tis N0M0), genetic counseling was ordered in accordance with National Comprehensive Cancer Network (NCCN) guidelines. Our patient qualified to have BRCAPlus testing by Ambry Genetics, which evaluated for the presence of six high-risk breast cancer genes. The analysis revealed a mutation in the breast cancer 2 (*BRCA2*) gene (p.Q1416). This finding was highly unsuspected because the patient did not have a family history of breast cancer for three generations prior, and all family members on both the maternal and paternal side lived to the ages of 80 years (Figure 5). Simultaneously, while she was being evaluated for a genetic mutation, magnetic resonance imaging (MRI) was obtained to screen the right breast for any abnormalities, as well as identify any gross invasive disease in the left breast. On MRI (Figure 6), the right breast showed a 5 mm nodule suspected to be an intramammary lymph node. The left breast was noted to have an extensive abnormal clumped nodular enhancement with a possible 1 cm mass suspicious for invasive disease. A follow-up ultrasound-guided core biopsy of the left breast 1 cm mass identified a fibroadenoma with high-grade DCIS.

**Figure 7:**
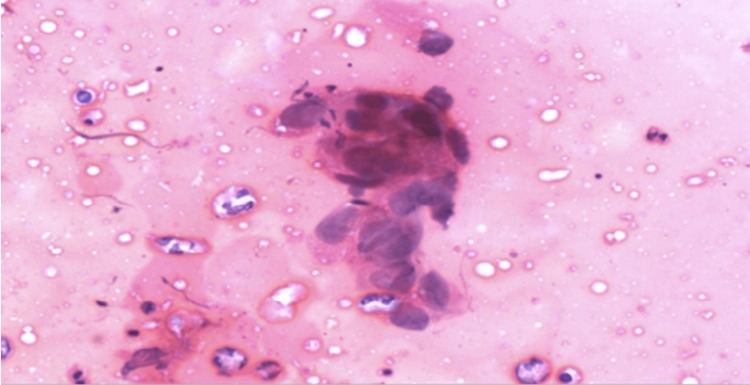
Cytology imprint using hematoxylin and eosin stain, showing characteristic Paget cells.

Extensive discussion was carried out with the patient regarding the recommendation to proceed with a bilateral mastectomy due to her known *BRCA2* mutation; however, she requested imprint cytology of the left nipple to confirm nipple involvement. She was advised against the cytology imprint because of the high risk of obtaining a false-positive result. In addition, a positive result would not have changed our recommendation for a left breast mastectomy with sentinel lymph node (SLN) biopsy. Ultimately, a cytological imprint of her left eczematous NAC was performed and confirmed Paget's disease of the breast (Figure 7). As seen in the image, the characteristic Paget cells were identified. These are often described as large cells with irregular nuclei and predominant nucleoli, surrounded by a large and pale cytoplasm. These cells can be seen either alone or in clusters.

After careful evaluation regarding treatment options, our patient elected to proceed with a left breast skin-sparing simple mastectomy with sentinel lymph node biopsy and immediate tissue expander placement for implant reconstruction. Although a prophylactic right breast mastectomy was recommended, the patient refused this suggestion. To address the disparity of the MRI abnormality on the right side, our patient agreed to proceed with a wire-localized excisional biopsy of the right breast mass, suspected to be 5 mm intramammary lymph node (too close to the skin for a needle biopsy at the time of the definitive left breast surgical procedure).

**Figure 8:**
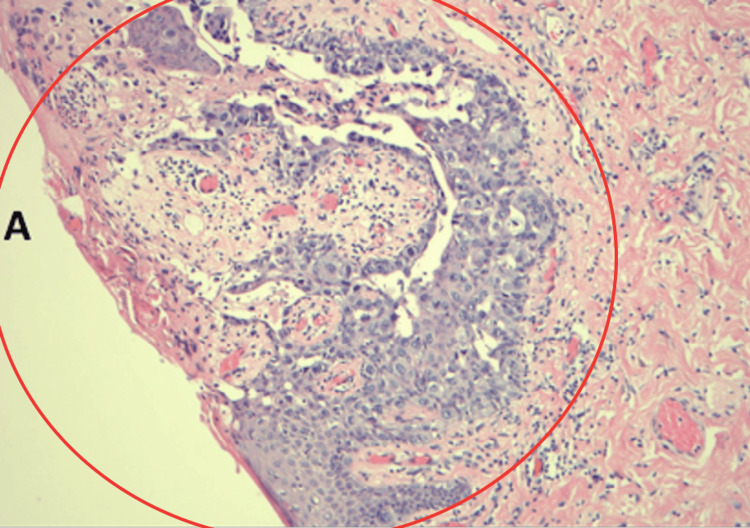
Hematoxylin and eosin (H&E) staining of the surgical specimen postmastectomy involving a section of the left nipple and breast indicating Paget's disease and DCIS. DCIS: ductal carcinoma in situ

**Figure 9:**
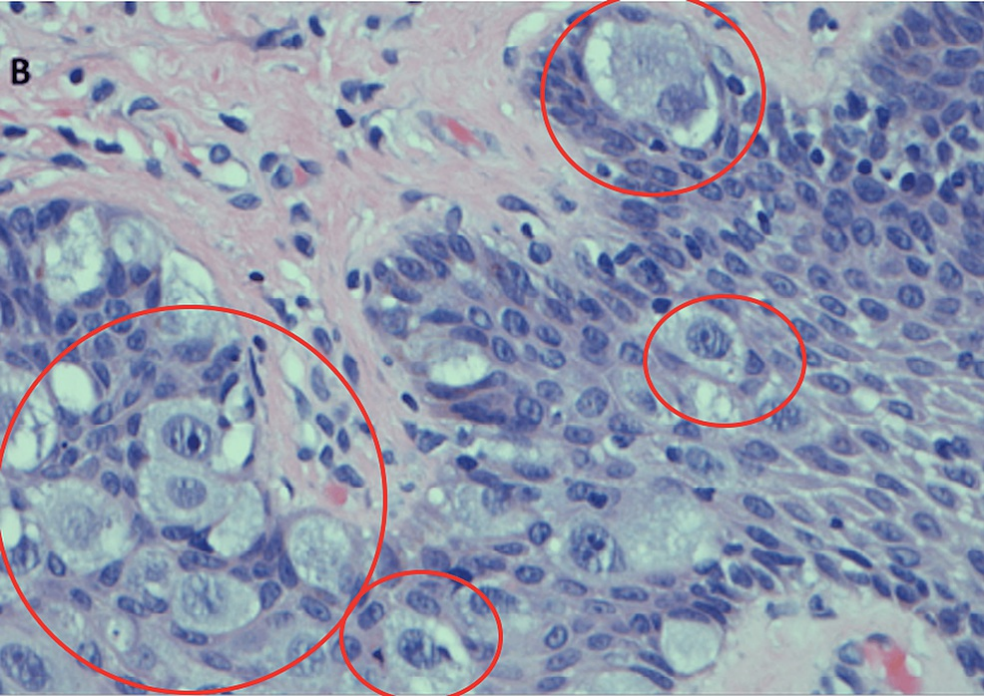
Magnification of H&E staining demonstrating Paget's disease. H&E: hematoxylin and eosin

The patient recovered well after surgery. Histological stainings (Figures 8, 9) and the final pathology report revealed left breast high-grade DCIS without invasion for an extent of 16 cm and ER and PR of 0%, with two negative sentinel lymph nodes (SLN). The right breast biopsy was found to be an intramammary lymph node without evidence of malignancy. She now remained as a pathologic stage 0 Tis N0M0. No adjuvant radiation therapy was recommended given the negative margins. Our patient was advised by our medical oncologist to be treated with tamoxifen to reduce the chance of new cancer in her right breast. She also agreed to right breast short-interval surveillance with an annual mammography and MRI with the possibility of considering prophylactic surgery along with oophorectomy after childbearing.

## Discussion

Paget's disease of the breast represents an uncommon disorder with an associated underlying breast cancer more than 65% of the time [2,6,7]. Almost exclusively, the underlying breast cancer is found to be either DCIS or invasive ductal carcinoma with the invasion of the epidermis by Paget cells [6]. As seen with our patient, despite the well-associated NAC changes and underlying malignancy, Paget's disease can remain undiagnosed for long periods of time and is frequently treated with topical steroid creams for dermatitis or eczema upon initial consult. Once the topical medications fail to resolve the symptoms, the differential diagnosis tends to shift away from infection or dermatitis and starts to focus on malignancy. It remains unanswered whether or not nipple cytology performed at our patient's initial visit would have saved the additional two-month delay in her treatment. Although a quick and easy procedure, breast surgeons have refrained from its use due to its low reported sensitivities for the detection of malignancy. However, a recent study described a sensitivity as high as 85%, suggesting a valuable utility for the earlier diagnosis of diseases such as Paget's disease [8].

National Comprehensive Cancer Network (NCCN) guidelines recommend bilateral diagnostic mammograms in patients older than 30 with breast symptoms. Diagnostic mammograms for females younger than 30 are considered low yield because of the density of the breast and the low risk for breast cancer and the high risk of false positives. Therefore, females under 30 years old with breast cancer often undergo ultrasound imaging. In our patient's case, given the mammographic abnormality of diffuse suspicious calcifications and a two-site stereocore biopsy establishing a diagnosis of underlying multicentric DCIS, we did not need a nipple biopsy. Cytology testing was only performed to prove to the patient that her nipple could not be salvaged.

Although Paget's disease typically presents during the fifth to sixth decade of life, literature has identified a patient as young as 24 years old with this disease [9]. The patients presenting with such a young onset of breast cancer usually have a family history of either first-degree breast or ovarian cancer with a known germ-line mutation. Studies indicate that 5% of breast cancer cases occur as part of a genetic susceptibility syndrome, and of those, 16% can be attributed to either a breast cancer 1 (*BRCA1*) or *BRCA2* germ-line mutation [9]. In addition, invasive ductal carcinoma is the most common histological type in *BRCA2* tumors (76%) [9]. Thus far, our report is the only one in the current literature of a patient who not only presented with a rare young-age diagnosis of Paget's disease but also had a sporadic rather than hereditary *BRCA2* mutation. In addition, no studies to our knowledge have identified an association between Paget's disease and either of the *BRCA* mutations (*BRCA1* and *BRCA2*).

In regard to treatment, the literature indicates that bilateral prophylactic mastectomies reduce the breast cancer risk by 90%-95% in females at increased breast cancer risk [4]. However, in our case, our patient elected to perform a left breast mastectomy only and did not desire to proceed with a prophylactic right breast mastectomy. She opted to be monitored carefully with an annual mammography and MRI. This is worrisome because a recent study found that young age at diagnosis is also associated with an increased risk for contralateral breast cancer. Patients ≤ 35 years have a 1.8-fold increased risk relative to females > 36 years of age [6]. Moreover, in females with *BRCA1* or *BRCA2* mutations, the current literature estimates a lifetime risk of breast cancer contralateral (CBC) that exceeds 40% [6]. In our case, our patient meets two high-risk factors for CBC; she was not only younger than age 35 at the time of cancer diagnosis but also *BRCA2-*positive. As such, a strong consideration of prophylactic management and treatment is required for patients with an increased risk of CBC.

The surgical decision-making process is highly personal and based on complicated therapeutic concerns. It is difficult to convince younger patients to undergo prophylactic bilateral mastectomies when diagnosed with genetic mutations because it significantly and permanently impacts their quality of life. In cases where cancer is diagnosed in patients with *BRCA1* or *BRCA2* mutations, options for treatment include breast-conserving therapy (BCT, defined as partial mastectomy and radiotherapy) or mastectomy along with contralateral prophylactic mastectomy. For those who decline contralateral prophylactic mastectomy treatment, chemoprevention along with systemic adjuvant treatment based on tumor biology could be advised with high-risk surveillance. This is important since the literature states that *BRCA1* or *BRCA2* mutation carriers may have an increased sensitivity to chemotherapy [6]. However, a study by Pierce et al. found "increased rates of local failure following BCT when compared with mastectomy but no difference in distant failure or disease-specific or overall survival between BCT and mastectomy" [6]. As such, the decision to undergo a prophylactic contralateral mastectomy is difficult and complicated. Every factor and management option should be taken into consideration when discussing treatment with patients afflicted by Paget's disease, especially those with *BRCA* mutations.

## Conclusions

Paget's disease of the breast is a rare cancer that is better known to affect females who are 60-70 years old. It can present clinically in various ways, including asymptomatically, as an eczematous rash, or as nipple pruritus. Although the literature has shown the ability of nipple cytology to diagnose Paget's disease, the diagnosis is typically made by nipple biopsy. Once identified, the acceptable methods of treatment include mastectomy or breast conservative treatment (criteria of <5 cm extent and unicenteric). However, because *BRCA* mutation carriers have a greater risk of both ipsilateral and contralateral breast cancer, high consideration should be given to performing a bilateral mastectomy to reduce their risk of a second breast cancer, despite being acceptable candidates for BCT. Moreover, *BRCA* patients should be advised on bilateral salpingo-oophorectomy to minimize the risk of ovarian cancer, especially if the patient has completed childbearing.
